# miRNAs in Normal and Malignant Hematopoiesis

**DOI:** 10.3390/ijms18071495

**Published:** 2017-07-11

**Authors:** Ryutaro Kotaki, Ryo Koyama-Nasu, Natsuko Yamakawa, Ai Kotani

**Affiliations:** Department of Hematology and Oncology, Tokai University School of Medicine, Hiratsuka 259-1193, Japan; kotaki-r@k-lab.jp (R.K.); ryonasu@tokai.ac.jp (R.K.-N.); natsukoyamakawa1984@gmail.com (N.Y.)

**Keywords:** miRNA, lymphoma, leukemia, B-cell

## Abstract

Lineage specification is primarily regulated at the transcriptional level and lineage-specific transcription factors determine cell fates. MicroRNAs (miRNAs) are 18–24 nucleotide-long non-coding RNAs that post-transcriptionally decrease the translation of target mRNAs and are essential for many cellular functions. miRNAs also regulate lineage specification during hematopoiesis. This review highlights the roles of miRNAs in B-cell development and malignancies, and discusses how miRNA expression profiles correlate with disease prognoses and phenotypes. We also discuss the potential for miRNAs as therapeutic targets and diagnostic tools for B-cell malignancies.

## 1. Introduction

Blood is composed of cells from multiple lineages, including myelocytes, lymphocytes, monocytes, platelets, and erythrocytes. Such cells play critical roles in multiple physiological functions, including generating immune responses, supplying oxygen to tissues, and maintaining hemostasis. Blood cells are continually generated from hematopoietic stem cells (HSCs) through a multi-step cell differentiation process called hematopoiesis. Each cell type has been characterized at specific developmental stages and the mechanisms of lineage specification have been extensively investigated during hematopoiesis. Notably, each step of hematopoiesis is regulated by a highly integrated network of transcription factors and microRNAs (miRNAs) [1,2,3].

In this review, we discuss the regulation of hematopoiesis by miRNAs, with a particular focus on B-cell differentiation. We also discuss the association between dysregulation of hematopoiesis and different blood cancers. The involvement of miRNAs in hematopoiesis might be used as a therapeutic alternative to treat blood cancers.

## 2. Hematopoiesis and B-Cell Development

Blood cells differentiate from HSCs through multiple stages, including multi-potent progenitors (MPPs), and restricted or committed progenitors, the fates of which are restricted at each step of hematopoiesis. A classical model of hematopoiesis describes the well-ordered step-wise lineage restrictions, including the differentiation of MPPs into lymphoid-restricted common lymphoid progenitors (CLPs) or myeloid and erythroid-restricted common myeloid and erythroid progenitors. In this context, only CLPs differentiate into B cells or T cells but not into myeloid or erythroid cell types, and vice versa.

Studies by Katsura’s group revealed a complex process in hematopoietic lineage restriction [4,5,6] in which progenitor cells differentiated into T myeloid cells or B myeloid cells at the single cell level, while no restricted T or B progenitor cells were apparent. Based on that data, a myeloid-based model for hematopoiesis was proposed [7]. Other groups also identified T-, B-, and myeloid-restricted progenitor cells, which is inconsistent with the classical model [8,9,10,11].

Previous studies revealed a complex network of transcription factors involved in the differentiation of lineages [1,2,3]. In B-cell development, PU.1, E2A, EBF1, and Pax5 are involved in lineage commitment [12,13,14,15]. Additionally, V (D) J recombination, which is a unique mechanism of genetic recombination that occurs in developing lymphocytes during the early stages of T- and B-cell maturation, is tightly regulated by transcription factors.

Mature B cells migrate to secondary lymph tissues, including the lymph nodes and spleen. Upon antigen stimulation, B cells relocate to the germinal center (GC), where they and helper T cells are activated. Activated mature B cells in the GC induce somatic hypermutation and class switch recombination, which involve genomic alterations. The activated B cells ultimately differentiate into plasma cells that produce antibodies and memory B cells.

## 3. miRNAs in B-Cell Differentiation

In addition to transcription factors, recent studies have identified miRNAs as regulators of gene expression. miRNAs are a class of 18–24 nucleotide (nt)-long noncoding RNAs that post-transcriptionally downregulate target genes. Primary miRNA transcripts are processed by the nuclear nuclease Drosha into ~60 nt precursor miRNA hairpins that are cleaved by the Dicer nuclease in the cytosol, generating mature miRNAs. Mature miRNAs are incorporated into the multiprotein RNA-induced silencing complex (RISC), which consists of Argonaut proteins (Ago). RISC post-transcriptionally represses target mRNAs by inducing their degradation or blocking translation [16,17,18]. Each miRNA has multiple target mRNAs, and in silico predictions suggest that more than one-third of all human genes are targets of miRNAs [19]. In animals, miRNAs regulate multiple developmental and physiological processes. For example, abnormal expression of specific miRNAs, including let-7 and lin-41, leads to developmental arrest in *C. elegans* [20].

miRNAs are also involved in the sequential differentiation of B cells, which was revealed in studies involving Ago2-deficient mice or mice lacking Dicer [21,22]. Ago2 is the primary component of the RISC protein complex, in which miRNAs bind target RNAs and prevent translation. Four Ago-family proteins exist in mammals; however, only Ago1 and Ago2 are expressed in hematopoietic cells (unpublished data). Therefore, loss of Ago2 largely inactivates the miRNA machinery during hematopoiesis.

Dicer performs the final step in miRNA processing, and thus, its loss prevents miRNA maturation. Notably, mice deficient in Ago2 and Dicer fail to convert pro-B cells into pre-B cells, which is critical for B-cell lineage commitment [23]. Multiple miRNAs, including miR-17–92, miR-34a, miR-125b, miR-150, miR-181a, and miR-212/132, regulate early B-cell development [24,25,26,27,28,29,30,31]. The miR-17–92 cluster and miR-181a positively regulate B-cell development, whereas the others negatively regulate the process. The miR-17–92 cluster is essential for the pro- to pre-B cell transition, since it prevents expression of the pro-apoptotic protein BIM [24]. Ectopic expression of miR-181a enhanced B-cell differentiation [30], while miR-34a negatively regulated B-cell development by downregulating FOXP1 [25].

Ectopic expression of miR-125b skewed hematopoiesis toward the myeloid lineage, whereas B-cell differentiation was decreased [27]. This finding was likely due to the repression of *LIN28A* by miR-125b. Ectopic expression of miR-150 impaired B-cell development [29]. B1-cell differentiation is also regulated by miR-150. The transcription factor c-Myb is a target of miR-150, and its repression may be responsible for those observations. The miR-212/132 cluster suppresses B-cell differentiation at the pre-B to pro-B transition stage by repressing *SOX4* [31]. Thus, multiple miRNAs are involved in B-cell development.

During late B-cell maturation in follicles, the downregulation of miR-150 is required for GC selection and development of the adaptive humoral immune response [29]. Other miRNAs, including miR-155, miR-181b, miR-15a, miR-16, miR-15b, miR-34a, miR-9, miR-30, let-7a, miR-125b, miR-217, and miR-185, modulate the expression of genes involved in B-cell maturation [32]. The GC is a transient structure that forms within the peripheral lymphoid organs in response to B-lymphocyte stimulation. Zhang et al., categorized mature B cells into naïve, memory, GC, and plasma cells and found that miR-223 was downregulated specifically in GC-B cells. miR-223 targets LMO2, which is abundant in GC-B cells. Conversely, miR-30 and miR-9, which target the PRDM1 transcription factor, were upregulated in GC-B cells, in which PRDM1 was found to be critical for plasma cell differentiation [33]. Malpeli et al., reported that of 48 miRNAs differentially expressed in naïve, GC, and subepithelial B cells, eight (miR-323, miR-138, miR-9*, miR-211, miR-149, miR-373, miR-135a and miR-184) were specific to follicular cells [34]. miR-125b also repressed the expression of *Prdm1* and *Irf4*, which encode transcription factors involved in plasma cell differentiation [26,27]. Additionally, miR-125b targeted Bright/*ARID3a*, which encodes a transcription factor involved in the expression of Ig heavy chains and B1-cell differentiation [28,35,36].

## 4. miRNAs in B Cell Malignancy

### 4.1. General View of miRNAs in Cancer

Multiple studies have correlated changes in miRNA expression profiles with human tumor phenotypes [35,36]. The first studies on the roles of miRNAs in cancer were of miR-15 in chronic lymphocytic leukemia (CLL). Croce et al., found that miR-15a and miR-16-1 are located at the 13q14 locus, which was deleted in the majority of CLL cases [37]. Since then, many miRNA have been revealed to have unique functions in cancer biology. For example, the expression of let-7 family members, which downregulate the expression of *Ras* and other proto-oncogenes, was reduced in lung cancer [38,39]. In several B cell malignancies, including acute lymphoblastic leukemia (ALL) and lymphoma, let-7 showed tumor suppressive functions [40,41]. Additionally, miR-15 family members, including miR-16, which was originally identified in CLL, a B cell malignancy, and miR-195, harbor tumor-suppressive functions by downregulating *BCL2* in several cancers. miR-200 suppresses the epithelial-mesenchymal transition, which is involved in metastasis [42,43]. In gastric diffuse large B cell lymphoma (DLBCL), miR-200 is tumor suppressive [44].

**Figure 1:**
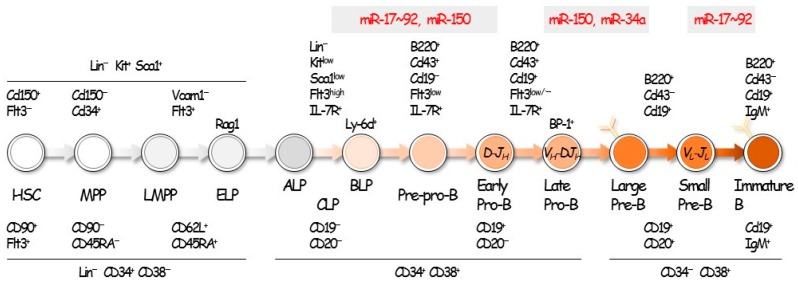
B-cell development and miRNAs. B-cell development is regulated by multiple transcription factors and miRNAs. The expression of miRNAs differs during each stage of B-cell development, and the dysregulation of specific miRNAs impairs B-cell development, causing tumor formation.

In contrast, the expression of miR-21, miR-17–92, and miR-155 is typically increased in cancers [45], suggesting their roles as oncogenes [46,47]. miRNAs expression, similar to that of protein-coding genes, is regulated by multiple transcriptional networks as well as the epigenetic machinery. In addition, miRNAs can themselves repress key enzymes that drive epigenetic remodeling, generating regulatory circuits that have a significant effect in the transcriptional landscape of the cell. Recent evidence also suggests that miRNAs can directly modulate gene transcription in the nucleus through the recognition of specific target sites in promoter regions. These mechanisms are also involved in the expression of tumor suppressive and oncogenic miRNAs [48]. Collectively, miRNAs play a primary role in cancer biology. Here, we focused on the miR-21, miR-34a, miR-150, miR-155, and the miR-17–92 clusters, which are involved in B-cell malignancies (Figure 1).

### 4.2. miR-21

miR-21 is one of the most abundant miRNAs in solid tumors. miR-21 is expressed in non-Hodgkin lymphomas. In diffuse large B cell lymphoma (DLBCL), miR-21 was found expressed at a higher level in ABC-type than in GC-type DLBCL [49]. miR-21 expression is correlated with the prognosis of CLL [50]. Rossi et al., performed quantitative reverse-transcription polymerase chain reaction (qRT-PCR) profiling in 104 CLL patients with a well-defined chromosome 17p status and found that miR-21 was differentially expressed between CLLs with a 17p deletion and those with a normal 17p karyotype. Moreover, miR-21 levels were significantly higher in patients with a poor prognosis and were correlated with predictions of overall survival (OS). Accordingly, a 21FK score (miR-21 qRT-PCR, fluorescence in situ hybridization, karyotype) was developed to stratify patients according to OS. Importantly, studies found that patients with a low score had a significantly longer OS. Evaluations of the power of the 21FK score with common prognostic factors revealed that the score was most significant in both CLL cohorts. Thus, 21FK score is useful for distinguishing between good and poor prognoses in CLL patients [50].

miR-21 is induced by EBNA2, a protein encoded by the Epstein-Barr virus (EBV) genome that causes cell proliferation though phosphorylation of AKT [51]. One of the targets of miR-21 is PTEN, which encodes a tumor suppressor [52]. Exogenous expression of miR-21 induced pre-B-cell leukemia [53], while suppression of miR-21 caused apoptosis and tumor regression. miR-21 knockdown in DLBCL cells increased their sensitivity to cyclophosphamide, vincristine, Adriamycin, and prednisone (CHOP) chemotherapies [52]. Knockdown of NF-κB also induced the same effect in DLBCL cells. Genotoxic treatments upregulated oncogenic miR-21 expression by recruiting NF-κB to the miR-21 promoter, where it activated the signal transducer and activator of transcription 3 [54]. miR-21 regulates NF-κB through several pathways, including extracellular signal-regulated kinase (ERK) [55]. Those observations suggest that NF-κB and miR-21 formed a positive feedback loop.

### 4.3. miR-34a

miR-34a is a tumor suppressor whose expression is regulated by another tumor suppressor, p53. Downregulation of miR-34a is correlated with a poor prognosis in CLL, DLBCL, and mantle cell lymphoma (MCL). Exogenous expression of miR-34 downregulated MYB and E2F1 and caused cell-cycle arrest. miR-34a regulates AXL, which encodes a receptor, tyrosine kinase, overexpressed in CLL [56]. MYC negatively regulates miR-34a expression by binding to its promoter. Notably, miR-34a was downregulated in lymphomas with high levels of MYC, while it was also found to regulate many targets of MYC [57]. The details of this contradiction have not yet been elucidated. The downregulation of miR-34a also induced FOXP1 and BCL6, which resulted in proliferation of DLBCL [57].

### 4.4. miR-150

miR-150 is a tumor suppressor that is downregulated in DLBCL, MCL, aggressive CLL, and Burkitt lymphoma (BL) cells [58,59]. Rescue of miR-150 in BL cells suppresses cell proliferation [58]. The targets of miR-150 are MYB, GAB1, and FOXP1, which affect B-cell receptor (BCR) and AKT signaling [59]. GAB1 acts upstream of BCR signaling and juxtaposes phosphoinositide 3-kinase (PI3K) at the plasma membrane. FOXP1 regulates the expression of multiple genes upon activation of BCR signaling, and overexpression of FOXP1 was correlated with a poor prognosis in CLL, DLBCL, and follicular lymphomas (FLs) [59]. Overexpression of FOXP1 in B-cell lymphomas is caused by downregulation of miR-34a and miR-150.

The chemokine receptor CXCR4 is also a target of miR-150. Overexpression of CXCR4 is caused by downregulation of miR-150 and activates the invasion of tumor cells [60]. However, the mechanisms underlying miR-150 regulation are unknown.

### 4.5. miR-155

miR-155 overexpression occurred in DLBCL, CLL, FL, and MCL [61,62,63,64,65]. The ABC-type DLBCL had a poor prognosis and was associated with increased expression of miR-155. E(mu)-miR-155 transgenic mice developed malignant B-cell lymphomas [47], and miR-155 was involved in lymphomagenesis by repressing IL-6 signaling, which resulted in dysregulation of B-cell differentiation [66,67]. miR-155 also repressed Smad5, which suppressed TGF-beta signaling and increased cell proliferation [68,69,70,71]. Additionally, miR-155 facilitated cell migration by downregulating HGAL, which may contribute to malignant tumorigenesis [72]. Iqbal et al., identified predictive miRNA biomarker signatures in DLBCL, including miR-155, which was significantly associated with rituximab plus CHOP treatment failure [73].

miR-155 is regulated by NF-κB. LMP1, which is encoded by the EBV genome, activates NF-κB signaling and induces the expression of miR-155 in EBV-infected B-cell malignancies [74]. NF-κB-induced overexpression of miR-155 also plays a role in ABC-type DLBCL. Thus, since miR-155 is involved in B-cell lymphomagenesis, it may be a promising therapeutic target.

### 4.6. miR-17‒92 Cluster

The miR-17‒92 cluster is comprised of six miRNAs, including miR-17, miR-18a, miR-19a, miR-20a, miR-19b, and miR-92a. This cluster facilitates formation of B-cell lymphomas in E(mu)-c-MYC transgenic mice, and its overexpression is involved in the formation of malignant B-cell lymphomas. Exogenous expression of the cluster induced development of DLBCL and CLL, suggesting that erroneous expression of miR-17‒92 causes lymphomas [75].

Overexpression of miR-17‒92 caused B-cell lymphomas as a result of downregulation of PTEN and BIM. Enforced expression of the mir-17–92 cluster acted with c-MYC expression to accelerate tumor development in a mouse B-cell lymphoma model [46].

MYC upregulated miR-17‒92 and E2F1, whereas miR-17 and miR-20a suppressed apoptosis by regulating E2F1 [76]. Recent studies revealed that the miR-17‒92 cluster activated the PI3K-AKT-mTOR pathway. miR-19a and miR-19b have also been found to be responsible for lymphomagenesis.

Intriguingly, miR-92a suppressed the function of miR-19. miR-92 induced MYC expression and p53-dependent apoptosis by suppressing FBW7, a ubiquitination enzyme targeting MYC [77]. Conversely, miR-19 counteracted miR-92-dependent apoptosis by activating MDM2, which targets p53 [78]. Depletion of the miR-17‒92 cluster exhibits more potent tumorigenesis. Collectively, the tumorigenic potency of the miR-17‒92 cluster is regulated by miR-19 and miR-92a.

## 5. Application of miRNAs in Diagnosis and Therapy

miR-21 and miR-155 are promising diagnostic markers for DLBCL and CLL [52,61,73,79,80,81,82]. Using in situ hybridization for formalin-embedded sections, such diagnostic markers will become simple to access [83,84]. Recent trials have used liquid biopsies to diagnose cancer during the early stages by use of exosome, which is stable in fluids such as blood, urine, and cerebral spinal fluid.

Exosomes contain a myriad of miRNAs [85,86]. Several miRNAs are promising diagnostic markers for several lymphomas; however, the methods of collecting and analyzing exosomes are variable. Thus, the development of standardized procedures for collecting and analyzing exosomes will be important for future studies.

Therapeutic trials using miRNAs have been performed for hepatitis C, and the greatest challenge reported by these trials was related to drug delivery [87,88]. Drug delivery is more complicated in hematopoietic cells compared with other cell types, including hepatocytes. Importantly, Ivan et al., reported that modified nucleic acids can be used to overcome drug delivery challenges in lymphomas. That study revealed that suppression of miR-155 in a mouse lymphoma model caused remission within one week [89]. Thus, miRNA regulation is one approach for drug delivery.

## 6. Concluding Remarks

miRNAs were discovered in mammals in 2000 and have since been linked to many cancers. Studies showed that miRNAs were more precise indicators of cancer phenotypes and prognoses than mRNAs. Those studies also presented miRNAs as promising diagnostic targets. Multiple researchers explored the potential of miRNA-based therapeutics. In the antisense approach to inhibit specific miRNAs, the antisense of miR-122, miravirsen, showed good results for HCV hepatitis on a phase2a trial. Miravirsen is a locked nucleic acid-modified DNA phosphorothioate antisense oligonucleotide that sequesters mature miR-122 in a highly stable heteroduplex, thereby inhibiting its function [88]. In the replacement approach to enhance the function of specific miRNAs, the replacement of miR-34 for advanced cancer is now on phase one trial [90]. Accordingly, miRNAs were promising targets for the diagnosis and treatment of B-cell malignancies.
